# Using Family-Based Imputation in Genome-Wide Association Studies with Large Complex Pedigrees: The Framingham Heart Study

**DOI:** 10.1371/journal.pone.0051589

**Published:** 2012-12-17

**Authors:** Ming-Huei Chen, Jie Huang, Wei-Min Chen, Martin G. Larson, Caroline S. Fox, Ramachandran S. Vasan, Sudha Seshadri, Christopher J. O’Donnell, Qiong Yang

**Affiliations:** 1 Department of Neurology, Boston University School of Medicine, Boston, Massachusetts, United States of America; 2 Department of Biostatistics, Boston University School of Public Health, Boston, Massachusetts, United States of America; 3 The National Heart, Lung, Blood Institute’s Framingham Heart Study, Framingham, Massachusetts, United States of America; 4 Wellcome Trust Sanger Institute, Hinxton, Cambridge, United Kingdom; 5 Center for Public Health Genomics, University of Virginia, Charlottesville, Virginia, United States of America; 6 Department of Mathematics and Statistics, Boston University, Boston, Massachusetts, United States of America; 7 Brigham and Women’s Hospital, Division of Endocrinology, Hypertension, and Diabetes and Harvard Medical School, Boston, Massachusetts, United States of America; 8 Preventive Medicine and Epidemiology, Boston University School of Medicine, Boston, Massachusetts, United States of America; 9 Cardiology Division, Department of Medicine, Massachusetts General Hospital, Harvard Medical School, Boston, Massachusetts, United States of America; University of Texas School of Public Health, United States of America

## Abstract

Imputation has been widely used in genome-wide association studies (GWAS) to infer genotypes of un-genotyped variants based on the linkage disequilibrium in external reference panels such as the HapMap and 1000 Genomes. However, imputation has only rarely been performed based on family relationships to infer genotypes of un-genotyped individuals. Using 8998 Framingham Heart Study (FHS) participants genotyped with Affymetrix 550K SNPs, we imputed genotypes of same set of SNPs for additional 3121 participants, most of whom were never genotyped due to lack of DNA sample. Prior to imputation, 122 pedigrees were too large to be handled by the imputation software Merlin. Therefore, we developed a novel pedigree splitting algorithm that can maximize the number of genotyped relatives for imputing each un-genotyped individual, while keeping new sub-pedigrees under a pre-specified size. In GWAS of four phenotypes available in FHS (Alzheimer disease, circulating levels of fibrinogen, high-density lipoprotein cholesterol, and uric acid), we compared results using genotyped individuals only with results using both genotyped and imputed individuals. We studied the impact of applying different imputation quality filtering thresholds on the association results and did not found a universal threshold that always resulted in a more significant p-value for previously identified loci. However most of these loci had a lower p-value when we only included imputed genotypes with with ≥60% SNP- and ≥50% person-specific imputation certainty. In summary, we developed a novel algorithm for splitting large pedigrees for imputation and found a plausible imputation quality filtering threshold based on FHS. Further examination may be required to generalize this threshold to other studies.

## Introduction

There are two main types of genotypic imputation for GWAS [1]. One type uses frequency and linkage disequilibrium (LD) information of a reference panel such as HapMap or 1000 Genome Project to impute the genotypes of genetic variants not included in the existing genome-wide genotyping. In the past few years, LD-based genotype imputation has been widely applied to GWAS that detected genetic associations for many complex human traits. Between November 2008 and January 2012, there were 252 publications that detected 2461 loci by using imputed genotype data according to the GWAS catalog (http://www.genome.gov/gwastudies).

The other type uses identity-by-descent (IBD) information in families to impute genotypes of un-genotyped individuals using the genotypes of their relatives. However, IBD-based genotype imputation has not frequently been applied to GWAS with family data. When phenotyped individuals exist who were not genotyped – perhaps due to limited genotyping resources or lack of a DNA sample – or poorly genotyped due to genotyping failure, poor quality DNA, or other reasons, IBD-based genotype imputation can be used to impute genotypes of these individuals and thereby increase sample size that potentially leads to better statistical power for genetic association studies.

Chen and Abecasis [2] developed an IBD-based imputation algorithm for GWAS, based on the Lander-Green [3] and Elston-Stewart [4] algorithms, which was implemented in their software package Merlin (http://www.sph.umich.edu/csg/abecasis/Merlin/) [5]. Based on 90 parents and grandparents of the Centre d’Etude du Polymorphisme Human pedigrees who were genotyped with 864360 single nucleotide polymorphisms (SNPs), they imputed the same set of SNPs for 78 offspring who were only genotyped with sparse genotypes (6728 SNPs). They observed an increase in power to detect association by including the imputed samples. Scuteri A. et al. [6] is another application of IBD-based imputation. Yet, the usefulness of IBD-based imputation has not been evaluated in studies with complex family relationships and with some individuals lacking any genotypes.

In the present investigation, we apply and evaluate the IBD-based imputation in the FHS that has recruited multiple generations of participants since 1948. The FHS sample consists of 14428 participants from 1538 pedigrees. Only 9274 have genotypes (Affymetrix 550K SNPs) and are part of the SNP Health Association Resource (SHARe). Among the rest, we imputed genotypes for those who have at least one genotyped relative using IBD-based imputation [2]. One challenge is that some large pedigrees exceed the computational limit of the software Merlin. Therefore, we propose a novel algorithm that uses kinship coefficients for splitting and trimming each large pedigree into multiple smaller sub-pedigrees and that can optimize the number of genotyped relatives for each un-genotyped individual in the sub-pedigrees.

After imputation, we evaluated how different imputation-quality filtering measures affected the results of GWAS with top SNPs for several phenotypes including Alzheimer disease, circulating levels of fibrinogen, high-density lipoprotein cholesterol (HDL) and uric acid. Using plausible imputation-quality thresholds, we conduct GWAS using the sample consisting of both genotyped and imputed individuals and compare with the GWAS results using genotyped individuals only.

## Results

### Splitting and Trimming Pedigrees

After splitting and trimming 122 pedigrees with bit size [7] (bit size = 2 * # non-founders – # founders – # un-genotyped founder couples) over 20, we obtained 629 sub-pedigrees. In the 122 pedigrees, there were 1187 un- or poorly- genotyped individuals with total 3068, 5405, and 3412, first, second, and third degree well-genotyped (call rate greater than 90% and heterozygous rate within +/−5 standard deviation range from mean) relatives, respectively. Poorly-genotyped individual is then defined as an individual with call rate not greater than 90% and heterozygous rate outside +/−5 standard deviation range from mean. In the 629 sub-pedigrees, 3060 (99.7%), 4431 (82.0%) and 1767 (51.8%) of the first, second, and third degree well-genotyped relatives, respectively, are retained.

### Genotype Imputation

**Figure 1:**
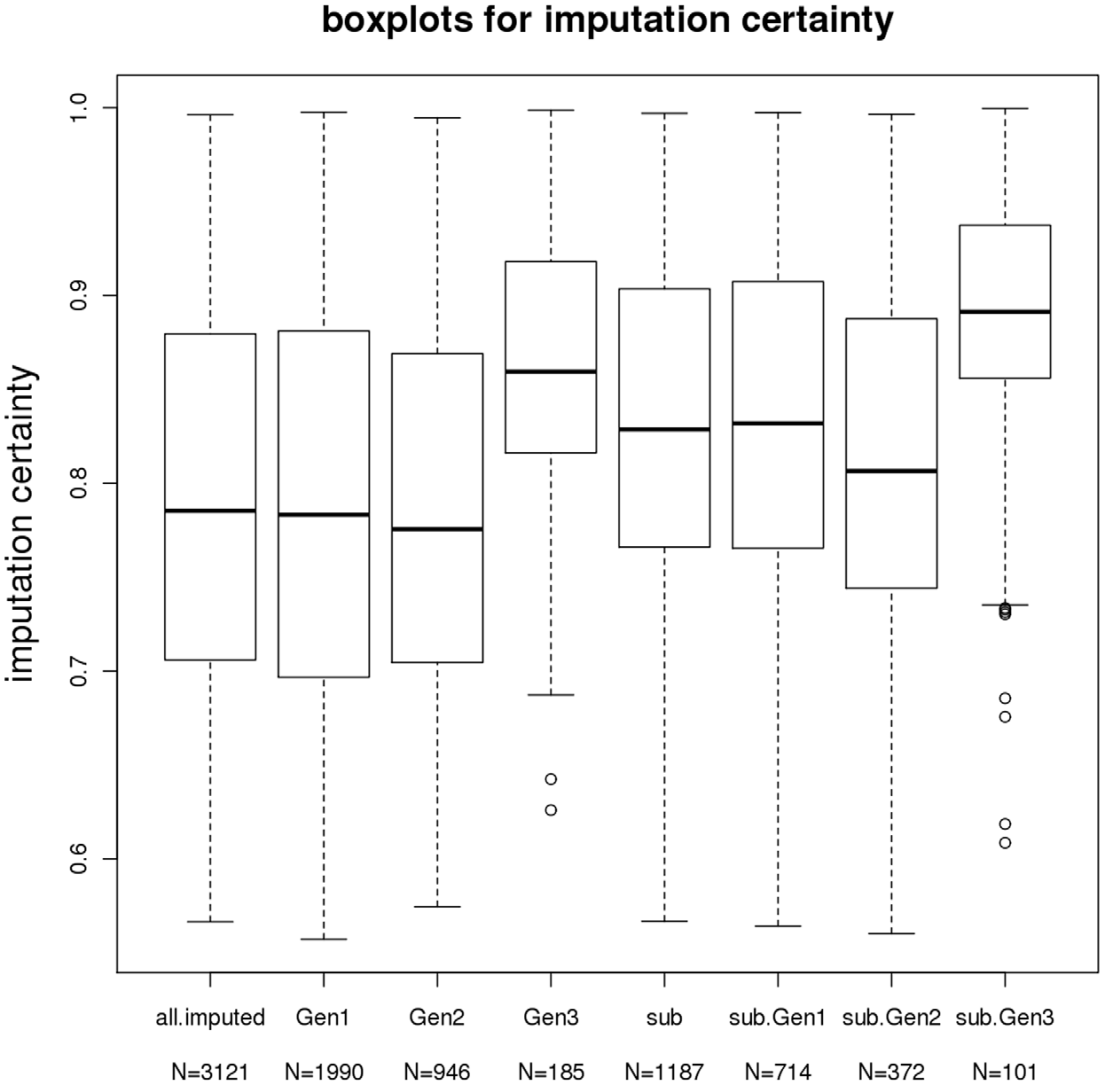
Box plots of imputation certainty in FHS imputed samples.

**Figure 2:**
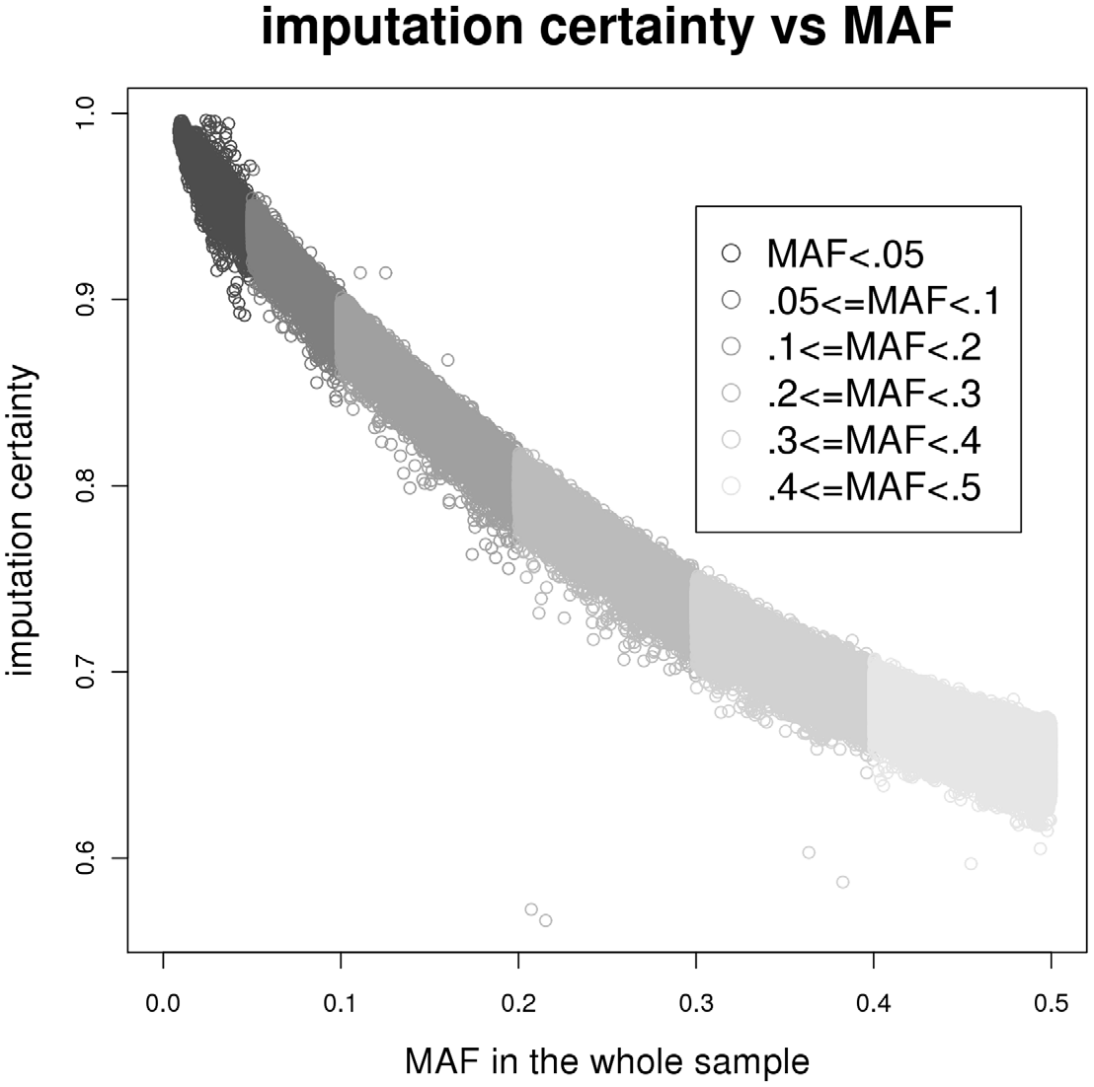
Scatter plot of imputation certainty against MAF.

**Figure 1** presents the box plots of average imputation certainty, the maximum of the posterior genotype probabilities of imputed genotypes, across all SNPs for the overall 3121 imputed individuals and the imputed 1187 individuals. Overall, the mean imputation certainty was 79.6% with standard deviation (SD) 9.9%. Among the overall imputed sample, the Third Generation sample had higher mean imputation certainty 86.8% and lower SD (6.1%) than the other cohorts. When comparing the imputation certainty from the all imputed sample to the 1187 sample in split sub-pedigrees, the sub-pedigree sample had a higher mean certainty 83.7% and smaller SD 7.9%. Similarly, for each generation, the mean generational imputation certainty in the 1187 sample is higher than the 3121 sample. **Figure** **2** presents the mean imputation certainty plotted against minor allele frequency (MAF). As expected, the imputation certainty decreased as the MAF increased, because when the MAF is low, most individuals are expected to carry the major allele homozygote, which translates to a high posterior probability for the major allele homozygote.

**Figure 3:**
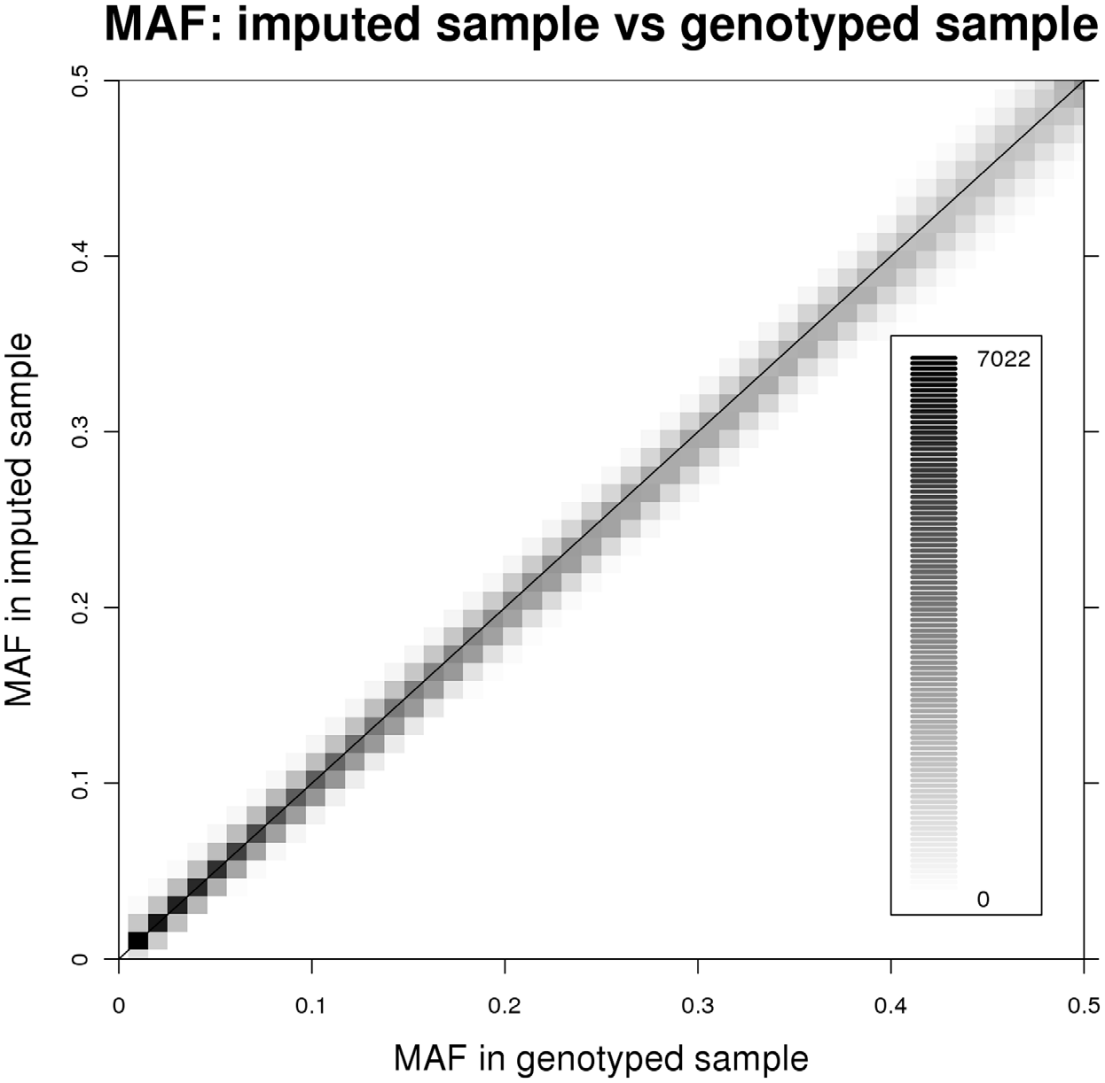
Scatter plot of MAF in well-genotyped sample and filtered (person_specific imputation certainty greater than 50%) imputed sample.

**Figure 3** presents the scatter plot of MAF in the filtered (person_specific imputation certainty greater than 50%) imputed sample (Y axis) against MAF in well-genotyped sample (X axis), where a cell represents the number of SNPs with MAFs that fall in that cell. When the number of SNPs in a cell increases, the color of the cell gets darker. Generally, data points are close to the 45 degree line. Among 403640 imputed SNPs, there are 23 and 368 SNPs with MAF difference (maximum 0.218) greater than 0.1 and 0.05, respectively, between well-genotyped and filtered imputed samples.

### GWAS with Genotyped Individuals

We first performed GWAS of Alzheimer disease, fibrinogen, HDL and uric acid including the 8998 well-genotyped individuals only. After filtering out SNPs with call rate less than 90%, HWE p-value less than 10^−6^ and MAF less than 0.05, we identified 8 independent loci (either on different chromosomes or at least 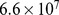 bps away from each other) using a genome-wide significance threshold of 1.25_×_10^−7^ (Bonferroni correction of 403640 SNPs). In addition, the locus close to *LIPC* on chromosome 15 for HDL – which did not reach genome-wide significance – was included because it reached genome-wide significance in the GWAS incorporating imputed individuals. The results for 9 selected loci are presented in **Table 1**.

**Table 1:** Top SNPs (p-value <1.25E-7) from GWAS of Alzheimer disease, fibrinogen, HDL and uric acid using 550K genotype data.

Trait	SNP	Chr	Position†	ClosestRefGene	HWE p	callrate	MAF*	N	beta	se	p
Alzheimer disease	rs4420638††	19	50114786	*APOC1*	0.78	0.999	0.16	3192	0.856	0.124	5.96E-12
Fibrinogen	rs4681	4	155710282	*FGB*	0.53	0.998	0.18	7271	10.009	1.446	4.48E-12
HDL	rs3764261	16	55550825	*CETP*	0.04	0.982	0.31	7996	3.077	0.266	5.71E-31
	rs1919484	8	19913956	*LPL*	0.11	0.981	0.27	7999	1.948	0.276	1.76E-12
	rs10186236	2	115096721	*DPP10*	0.21	0.999	0.19	8128	−1.647	0.307	7.79E-8
	rs1800588	15	56510967	*LIPC*	0.65	1.000	0.22	8134	1.514	0.293	2.42E-7
Uric acid	rs16890979	4	9531265	*SLC2A9*	0.01	0.998	0.25	8229	−0.352	0.022	2.64E-59
	rs2231142	4	89271347	*ABCG2*	0.76	0.999	0.11	8234	0.246	0.031	1.46E-15
	rs1165205	6	25978521	*SLC17A3*	0.19	0.985	0.46	8096	−0.105	0.019	4.34E-8

† Position in base pairs, based on NCBI build 36.1 (hg18).

* MAF is computed in genotyped and phenotyped sample.

†† rs4420638 is a marker of the APOE haplotype.

### Evaluate the Effects of Different Imputation Certainty Thresholds on GWAS Top Hits

Using GWAS top SNPs as positive controls, we evaluate how the association results change with various thresholds for person-specific certainty and SNP-specific certainty used to incorporate genotypes of imputed individuals with that of genotyped individuals. **Figures S1, S2, S3, S4** present the results (-log_10_ p-value) for Alzheimer disease, fibrinogen, HDL and uric acid. In each plot, the horizontal line presents the results from GWAS using genotyped individuals only. P-values from combined sample are more significant than that of GWAS with genotyped individuals only for most combinations of the person- and SNP-specific certainty thresholds, except for rs4681 (**Figure S2**) and rs10186236 (**Figure S3**). No combination of person- and SNP-specific certainty threshold gives uniformly best results; also, there is no clear relation between certainty thresholds and improvement in p-value.

To incorporate genotypes of imputed individuals with that of the genotyped individuals for GWAS, we choose the combination of person-specific certainty threshold 0.5 and SNP-specific certainty threshold 0.6 as a trade-off between quantity (sample size) and quality. This combination generally gives slightly better results than most of the other combinations based on our evaluations using those top SNPs. **Table 2** presents the mean imputation certainty for the top SNPs in the entire 3121 imputed sample and in the person-specific certainty >0.5, SNP_specific certainty >0.6 and phenotyped sample. By using these certainty thresholds, the median of the mean certainty improves from 0.81 (with minimum 0.66) to 0.95 (with minimum 0.93) and the average increased certainty is about 0.16. Of note, among the 3121 imputed individuals, there are 1481, 868, 467, and 116 individuals with person-specific certainty above 0.3, 0.5, 0.7, and 0.9, respectively.

**Table 2:** Mean imputation certainty of the top SNPs in the entire 3121 imputed sample and in the person-specific certainty >0.5, SNP-specific certainty >0.6 and phenotyped sample.

Trait	SNP	mean(sd) certainty in 3121 imputed sample	*N* †	mean(sd) certainty in *N*
Alzheimer disease	rs4420638	0.838(0.162)	288	0.955(0.074)
Fibrinogen	rs4681	0.824(0.161)	331	0.971(0.068)
HDL	rs3764261	0.742(0.187)	512	0.928(0.128)
	rs1919484	0.753(0.174)	524	0.930(0.117)
	rs10186236	0.802(0.167)	431	0.962(0.072)
	rs1800588	0.817(0.164)	419	0.951(0.092)
Uric acid	rs16890979	0.769(0.170)	595	0.939(0.100)
	rs2231142	0.884(0.142)	638	0.974(0.049)
	rs1165205	0.658(0.199)	553	0.980(0.068)

† ***N***: the number of phenotyped and imputed individuals with person-specific certainty >0.5 and SNP-specific certainty >0.6.

### GWAS Using Genotyped and Imputed Individuals

**Figure 4:**
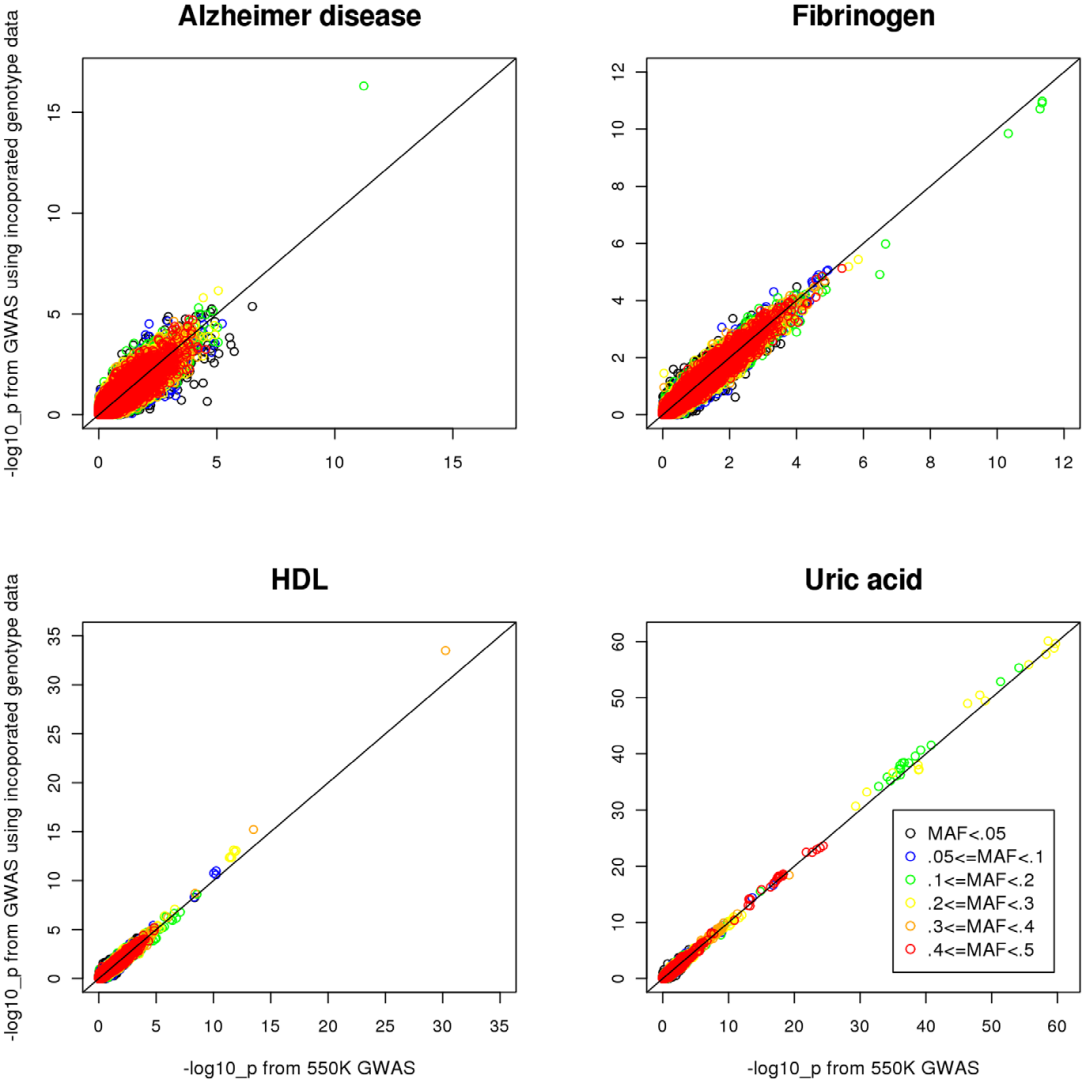
Scatter plots of –log_10_(p-value) from 550K GWAS and GWAS using incorporated genotype data.

We use the combination of person-specific certainty threshold 0.5 and SNP-specific certainty threshold 0.6 to combine genotype data of imputed with genotyped individuals. With the combined data, we redo the GWAS for Alzheimer disease, fibrinogen, HDL and uric acid, additionally adjusted for imputation status to account for the potential phenotypic difference between genotyped and imputed samples. The same filters (call rate <90%, HWE p-value <10^−6^ and MAF <0.05) are applied as in 550K GWAS, so we have the same SNPs for comparison. The results for the top SNPs and the genomic control parameter *λ* [8] from GWAS using genotyped sample and that of using combined imputed- and genotyped- sample are presented in **Table 3**. The *λ* estimates (1.02–1.03) show that no systematic inflation in test statistics is observed. The increase in sample size varies from about 300 to 600, which leads to slight decrease in the standard error estimate of the beta coefficient. Among the 9 independent loci, 7 loci improve their statistical significance after including imputed sample. **Table 4** presents the improvement in statistical significance level comparing the GWAS using the combined sample versus using genotyped sample only. Except for fibrinogen, most of the genome-wide significant SNPs have smaller p-values in GWAS using combined sample. In addition, for both HDL and uric acid, one additional SNP becomes genome-wide significant in GWAS using combined sample. **Figure 4** presents the –log_10_ p-value scatter plots of GWAS using combined sample (Y axis) against GWAS using genotyped sample (X axis). The figure shows that except for Fibrinogen, most of the SNPs that reach genome-wide significance have smaller p-values from GWAS using combined sample, as we reported.

**Table 3:** Results of top SNPs (p-value <1.25E-7) from GWAS of Alzheimer disease, fibrinogen, HDL and uric acid using 550K genotype data and incorporated genotype data.

		genotyped subjects only						genotyped and imputed subjects					
Trait	SNP	N	MAF	beta	se	p	*λ*	N	MAF	beta	se	p	*λ*
Alzheimer disease	rs4420638	3192	0.16	0.856	0.124	5.96E-12	1.02	3480	0.16	0.902	0.108	4.93E-17	1.03
Fibrinogen	rs4681	7271	0.18	10.009	1.446	4.48E-12	1.03	7602	0.18	9.729	1.431	1.05E-11	1.02
HDL	rs3764261	7996	0.31	3.077	0.266	5.71E-31	1.03	8508	0.32	3.155	0.259	3.15E-34	1.02
	rs1919484	7999	0.27	1.948	0.276	1.76E-12		8523	0.27	2.020	0.270	7.59E-14	
	rs10186236	8128	0.19	−1.647	0.307	7.79E-8		8559	0.19	−1.577	0.301	1.62E-7	
	rs1800588	8134	0.22	1.514	0.293	2.42E-7		8553	0.22	1.548	0.288	8.09E-8	
Uric acid	rs16890979	8229	0.25	−0.352	0.022	2.64E-59	1.03	8824	0.23	−0.351	0.021	8.13E-61	1.02
	rs2231142	8234	0.11	0.246	0.031	1.46E-15		8872	0.10	0.248	0.030	2.86E-16	
	rs1165205	8096	0.46	−0.105	0.019	4.34E-8		8649	0.47	−0.109	0.019	6.96E-9	

**Table 4:** Number of genome-wide significant SNPs (p-value <1.25E-7) with improved statistical significance (smaller p-value) from GWAS of Alzheimer disease, fibrinogen, HDL and uric acid using incorporated genotype data (new GWAS).

Trait	# SNPs with smaller p/# SNPs with p<1.25E-7 in 550K GWAS	# SNPs with p<1.25E-7 in new GWAS but not in 550K GWAS
Alzheimer disease	1/1	0
Fibrinogen	0/4	0
HDL	14/15	1
Uric acid	83/126	1

## Discussion

Using FHS sample, we have demonstrated that imputation in general improves statistical power for GWAS, even when the imputed individuals have not been genotyped at all. Using GWAS top hits identified with observed genotypes as positive controls, we explored the effects of different quality control thresholds for incorporating genotypes of imputed individuals on the association results. In order to perform imputation for large pedigrees that are too complex to be handled with realistic computing power, we developed an algorithm for splitting and trimming large pedigrees into sub-pedigrees that can optimizes the number of closest related genotyped relatives for each un- or poorly- genotyped individual. Unlike imputation of un-genotyped variants using external reference, family-based imputation is to impute un- or poorly- genotyped individuals. As these individuals do not have any genotypes or good quality genotypes, one cannot compute the actual imputation quality. Therefore imputation certainty is used as imputation quality measure.

The fact that the Third Generation cohort has better imputation certainty over the previous two generations is a result of more Third Generation individuals having at least one genotyped parent, or more genotyped relatives. The proportion of imputed Third Generation subjects having at least one parent is 91.9%, versus 0.2% and 32.3% for imputed individuals in the Original and Offspring cohorts, respectively. The average sum of genotyped 1^st^, 2^nd^ and 3^rd^ degree relatives per imputed individuals for the Original, Offspring and Third Generation cohorts among the 3121 imputed sample are 6.1, 5.3 and 9.2, respectively. In addition, the average sum of genotyped 1^st^, 2^nd^, and 3^rd^ degree relatives of the Original, Offspring and Third Generation cohorts among the 1187 individuals are 7.9, 7.1 and 9.8, respectively. This explains why the average imputation certainty is higher in 1187 individuals than in all 3121 individuals for each generation. The fact that the 1187 sample in split sub-pedigrees has higher mean imputation certainty than the rest imputed sample is due to imputed individuals in large pedigrees (thus need splitting) generally having more genotyped relatives, among whom, the most informative ones are retained in the split sub-pedigrees created using our algorithm. When regressing imputation certainty on the numbers of well-genotyped 1^st^, 2^nd^, and 3^rd^ degree relatives, the bit size and the number of members in sub-pedigree, the numbers of genotyped 1^st^ and 2^nd^ degree relatives and the number of members in sub-pedigree are positively associated with imputation certainty with p-values 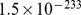, 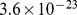, and 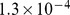, respectively. This indicates that most information is contributed by the 1^st^ degree genotyped relatives. As the proposed algorithm relies on the relationships within a large pedigree, results are sensitive to any pedigree misspecification in nature.

The imputation works well as we only have 23 and 368 SNPs with MAF difference (maximum 0.218) greater than 0.1 and 0.05, respectively, between well-genotyped and filtered imputed samples. The 23 SNPs have average 498 Mendelian errors, which suggests additional useful criterion for selecting SNPs for imputation. If the whole 3121 imputed sample is used, MAF will be in general underestimated. This reassures the necessity of using imputation certainty filter and the validation of our GWAS results using incorporated genotype data.

When incorporating imputed genotype data with observed genotype data, we consider various combinations of thresholds of person-specific certainty and SNP-specific certainty to filter out genotypes and individuals with lower imputation certainty. Although we have observed improved statistical significance for most combinations of thresholds for most of the top SNPs, there are still a few cases (rs4681 for fibrinogen and rs10186236 for HDL) with no improvement for any threshold combinations. **Table 2** indicates that failure to strengthen the statistical significance is not likely due to low imputation certainty, as the average certainties in incorporated imputed individuals for rs4681 and rs10186236 are 97.1% (top 3^rd^) and 96.2% (top 4^th^), respectively, and the improvement does not seem to be associated with high imputation certainty. The lack of improvement may be due to heterogeneity in phenotypes between imputed and genotyped individuals and/or noise in the imputed genotypes. Even though family-based imputation can increase the sample size that leads to power increase by theory, it also introduces noise due to the uncertainty in the imputed genotypes. There is no imputation certainty threshold combination that consistently gives better results than other combinations. The thresholds (person-specific certainty >0.5 and SNP-specific certainty >0.6) we have adopted for our sample seem working well. It may be applicable for other studies, but examination of the sensitivity is still warranted when applied to a different study. In addition, as shown in Figure 2, imputation certainty decreases as MAF increases. One can thus take MAF into consideration when applying SNP-specific imputation certainty threshold during quality filtering.

With both imputed sample (person-specific certainty >0.5 and SNP-specific certainty >0.6) and genotyped sample included in GWAS of Alzheimer disease, fibrinogen, HDL and uric acid, the statistical significance is strengthened for 7 out of 9 independent genome-wide significant loci, or for 98 out of 146 genome-wide significant SNPs, while the inflation measured by genomic control factor (*λ*) remains similar compared with GWAS using genotyped individuals only. Among the 7 loci, *APOC1* for Alzheimer disease has the smallest number in sample size increase (**Table 2**), but its proportion of reduction in standard error of beta is the largest and so is its increase in statistical significance (**Table 3**). In general one would expect the proportion of reduction in the standard error to be similar to the proportion of increase in the square root of sample size [9]. The disproportional change in this case is due to that Alzheimer disease is more common in the added imputed sample. There are 164 cases (5.1%) in 3192 genotyped individuals and 30 cases (10.4%) in 288 added imputed individuals. Except for the association between *DPP10* locus and HDL, all other associations have been previously reported or confirmed by meta-analysis [10]–[13]. **Figures S5, S6, S7, S8, S9, S10, S11, S12, S13** are the regional association plots by SNAP [14] for the 9 loci based on GWAS using incorporated genotype data. rs4420638 is 340 bp and 10297 bp away from *APOC1* and the well-known *APOE* genes, respectively. In FHS 550K data, no SNP is genotyped in *APOE* and no SNP is in high linkage disequilibrium with rs4420638. Therefore, as shown in **Figures S5** for incident Alzheimer disease, rs4420638 is the only genome-wide significant SNP in +/−200 kb region around itself. In addition, rs4420638 is strongly associated with *APOE* with p-value 3.3×10^−8^ as previously reported [12] in FHS. The association between rs1800588 (*LIPC*) and HDL becomes genome-wide significant after including the imputed sample. Similarly, the association between rs13148356 in *SLC2A9* and uric acid becomes genome-wide significant after imputation indicates that rs13148356 is also a likely truly associated variant missed by analyzing genotyped individuals only. The associations of rs4681 (*FGB*) with fibrinogen and rs10186236 (*DPP10*) with HDL are slightly weakened, but the latter association has not previously been reported.

Our results demonstrate that the proposed algorithm for splitting and trimming large pedigrees for IBD-based imputation worked well and that including the imputed sample with genotyped sample in GWAS generally strengthened the association signals for loci with associations that have already been well established. We identified a plausible imputation quality filtering threshold based on FHS. Further examination may be required to generalize this threshold to other studies.

## Materials and Methods

### Algorithm for Splitting and Trimming Large Pedigrees

The basic steps of the proposed algorithm for splitting and trimming large pedigrees are as follows: (i) form clusters of un-genotyped individuals with their closest (first degree) un-genotyped relatives; (ii) construct sub-pedigrees based on clusters; (iii) check the bit size of the sub-pedigrees; and (iv) apply trimming if the bit size is greater than desired. Details of the algorithm and a hypothetical example of applying the algorithm are described below.

For each pedigree that needs size reduction,

1. Clustering un-genotyped individuals
  1. Compute the number of un-genotyped first degree relatives for each un-genotyped individual.
  2. Form clusters by grouping un-genotyped first degree relatives together until all un-genotyped persons are clustered. We suggest starting from the un-genotyped individual with fewest un-genotyped first degree relatives. It is more efficient and can avoid large clusters that require more trimming that may leave insufficient genotyped individuals for imputation given a user specified bit size limit.
2. Constructing sub-pedigrees based on un-genotyped clusters
  1. Include genotyped blood relatives (kinship coefficient >0) of each cluster member.
  2. Include parents of current members (if not included).
  3. Remove un-genotyped founder couples that only have one child.
3. Checking bit size
  1. If bit size is less than but not close to user specified, add genotyped blood relatives of spouse. If bit size is greater than user specified, do trimming. Otherwise, sub-pedigree preparation is complete.
4. Trimming sub-pedigrees with bit size greater than user specified
  1. For each genotyped individual in a sub-pedigree, compute the following scores using kinship coefficients. A genotyped individual with more closest cluster members (score1 below) is more important for imputation and should not be trimmed if not needed. The order of importance of the scores is score1, score2, score3, score4, followed by score5.
  2.
    - score1: number of the closest cluster members (not necessarily first degree)
    - score2: number of the 2^nd^ closest cluster members
    - score3: number of the 3^rd^ closest cluster members
    - score4: number of the 4^th^ closest cluster members
    - score5: number of the 5^th^ closest cluster members
5.
  1. Identify roots (bottom level of the current sub-pedigree; roots should not be parents) that are not cluster members.
  2. Rank the roots by scores.
  3. Remove person or persons with the minimum rank
    - If a whole sibship is removed or only has one sib left, remove their founder-parents, too.
    - Compute bit size and repeat step 4.4 until bit size is less than user specified.
6.
  1. If bit size is less than user specified within 2 bits, trimming is done; otherwise, add back latest removed genotyped persons, so that the bit size is as close to and less than user specified.
  2. If a sub-pedigree is a subset of another sub-pedigree, remove it.

**Figure 5:**
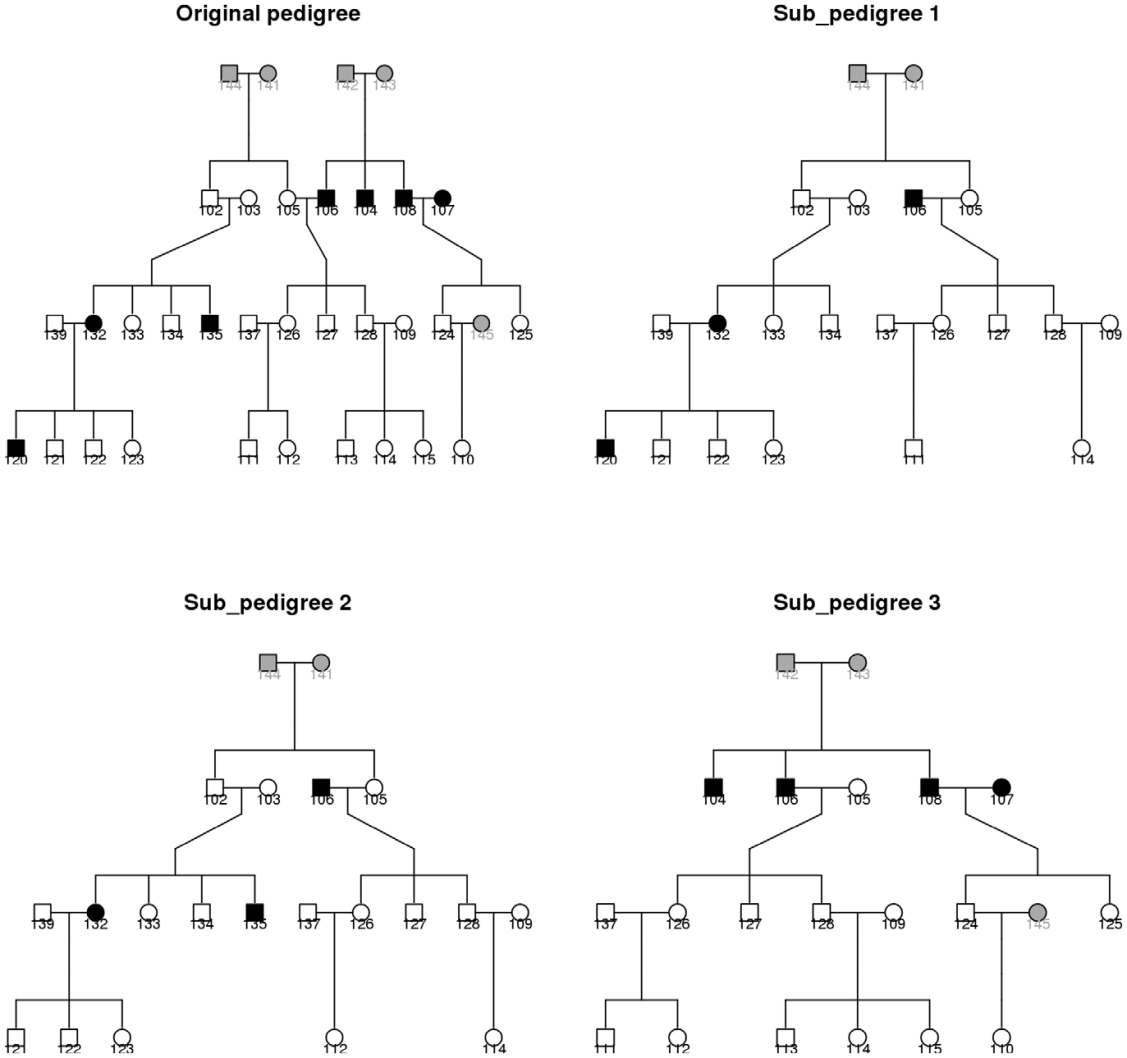
Example for pedigree splitting and trimming.

Example: Consider the example pedigree presented in **Figure 5** by kinship2 package (http://cran.r-project.org/web/packages/kinship2/), the original pedigree (top left panel) has 34 pedigree members and bit size of 36. A grey cell represents an individual not offering consent to participate in a study of interest, a black cell represents an individual to be imputed, and an empty cell represents a genotyped individual. The number under each cell is the individual ID. There are 7 individuals to be imputed in this pedigree (IDs 104, 106, 107, 108, 120, 132, 135) and they form 4 clusters in the following order, the 1^st^ by 107, the 2^nd^ by 132 and 120, the 3^rd^ by 135, the 4^th^ by 104, 106 and 108. The other 3 panels in **Figure 5** present the final 3 sub-pedigrees. Sub-pedigree 1 with bit size 20 is formed by the 2^nd^ cluster. Sub-pedigree 2 with bit size 20 is formed by the 3^rd^ cluster. Sub-pedigree 3 with bit size 20 is formed by the 4^th^ cluster and contains the sub-pedigree formed by the 1^st^ cluster. ID 132 appears in sub-pedigrees 1 and 2, as ID 132 belongs to the 2^nd^ cluster, genotypes imputed based on sub-pedigree 1 will be used for ID 132. The pedigree splitting and trimming scripts written in R are available on readers’ request.

Each resulting sub-pedigree is centered on one or more un-genotyped individuals with as many closely related genotyped relatives as possible. Between sub-pedigrees, there may be overlaps of genotyped individuals or un-genotyped individuals. The imputation is performed using one pedigree at a time; therefore, overlapping is not an issue. But the original pedigrees should be used in association analyses, not the split pedigrees.

### The Framingham Heart Study (FHS) Sample

The FHS was initiated in 1948 with the enrollment of 5209 men and women (referred to as the Original cohort) from Framingham, MA, who underwent biennial examinations [15]. In 1971, 5124 children and spouses of these children of the Original cohort (referred to as the Offspring cohort) were recruited and examined approximately every four years [16]. In 2002, 4095 Third Generation cohort participants were enrolled [17].

In 2007, genome-wide genotyping of SNPs was performed for 9274 individuals using an Affymetrix 550K SNPs platform; 8998 participants were well-genotyped, that is, call rate greater than 90% and heterozygous rate within +/−5 standard deviation range from mean. Among un-genotyped individuals and 276 poorly-genotyped (not well-genotyped) individuals, 3121 (from 928 pedigrees) with at least one genotyped blood relative and with consent for genetic studies, can be included for genotype imputation. The 3121 individuals included 1990, 946 and 185 Original, Offspring and Third Generation cohorts, respectively. All individuals included in this study provided written informed consent, and study protocols were approved by the Institutional Review Boards of Boston University. Merlin [5] was used in genotype imputation, and we found the smallest bit size of the pedigrees that Merlin failed to impute is 20. Therefore, we applied the proposed algorithm to split and trim the 122 pedigrees with bit size greater than 20 and used the split sub-pedigrees for imputation.

### Phenotype Definition and Measurement

The characteristics for each trait for the sample of genotyped and imputed individuals are presented in **Table 5**. Alzheimer disease was defined as previously described using NINCDS-ADRDA criteria [18]. Fibrinogen was measured in the Original cohort subjects during examination cycle 10 (1966–1968) using a modified method of Ratnoff and Menzie [19], in the Offspring cohort during examination cycle 5 (1991–1995) and in the Third Generation cohort during examination cycle 1 (2002–2005) using the Clauss method [20]. Serum urate was measured during the first examination cycle of each cohort using an autoanalyzer with a phosphotungstic acid reagent [21], and HDL was measured using standard enzymatic method in the Original cohort during examination cycles 11–13 (1970–76), in the Offspring cohort during examination cycle 6 (1996–2000) and in the Third Generation during examination cycle 1 (2002–2005).

**Table 5:** Sample characteristics of Alzheimer disease, fibrinogen, HDL and uric acid data in the genotyped and imputed sample.

	Alzheimerdisease	Fibrinogen(mg/dl)	HDL (mg/dl)	Uric acid(mg/dl)
Sample size	4200	8229	9453	10491
Phenotype	284 (6.8%)	321.2 (67.9)	52.6 (16.1)	5.3 (1.5)
Age	78.2 (8.2)	48.0 (12.0)	51.2 (13.2)	38.9 (9.8)
Sex (female)	2318 (55.2%)	4414 (53.6%)	5079 (53.7%)	5443 (51.9%)
Original cohort	1899 (45.2%)	1062 (13%)	2044 (21.6%)	1984 (18.9%)
Offspring cohort	2301 (54.8%)	3131 (38%)	3339 (35.3%)	4459 (42.5%)
Third Generation cohort	NA	4036 (49%)	4070 (43.1%)	4048 (38.6%)
Imputed	978 (23.3%)	942 (11.4%)	1313 (13.9%)	2248 (21.4%)
Length of follow-up (years)	13.2 (8.2)	NA	NA	NA

For continuous variables, mean value and standard deviation (in parenthesis) are presented, while for binary variables, the number of cases and its proportion (in parenthesis) are presented.

### Statistical Analyses, Genotype Imputation and Genotype Incorporation

GWAS of continuous traits (fibrinogen, HDL and uric acid) were performed using a linear mixed effects model with the additive coding of SNP genotypes as a fixed effect and with individual-specific random intercepts correlated according to the kinship coefficient to account for residual familial correlations [22]. Cox proportional hazards regression implemented in R survival package was used to model incident Alzheimer disease (starting at age 65 years); each pedigree was treated as a cluster and the robust variance estimate was used [23]. All analyses were adjusted for age, sex, generation status and imputation status if imputed individuals were included.

Based on the genotypes of 8998 well-genotyped individuals with Affymetrix 550K SNPs, we imputed genome-wide genotype data for the additional 3121 individuals (sparse Illumina Infinium panel genotyping of 5759 SNPs for 150 of them were also used in the imputation). Imputation was performed for 403640 autosomal SNPs with good genotyping quality, that is, call rate>0.97, MAF>0.01 and Hardy Weinberg Equilibrium (HWE) p-value>10^−6^, using split sub-pedigrees and original pedigrees with bit size not greater than 20. The imputed genotype dosage data is used in association analysis. The maximum of the posterior genotype probabilities from genotype imputation was used as an indicator of the imputation certainty at each SNP for each imputed individual, which we also called SNP-specific certainty.

For each individual, we compute the proportion of SNPs with the maximum posterior probability greater than 0.95, which is used to define person-specific certainty. Applying person-specific certainty threshold of 0.9 retains individuals with the proportion greater than 0.9. In contrast, applying SNP-specific certainty threshold of 0.6 retains individuals with imputation certainty greater than 0.6 for each SNP – and different individuals may be included for different SNPs.

To evaluate the effects of including imputed individuals in association testing, various quality filtering thresholds of imputation certainty (0, 0.3, 0.5, 0.7 and 0.9 for person-specific certainty, and 0.95, 0.9, 0.85, 0.8, 0.7, 0.6, 0.5, 0.4, 0.3, 0.2, and 0 for SNP-specific certainty) are considered to incorporate imputed individuals and their imputed genotypes with observed genotypes. Each incorporated genotype dataset is used to test the association of 8 GWAS top SNPs for Alzheimer disease, fibrinogen, HDL and uric acid that have been previously reported and serve as positive control. We then selected the incorporated genotype dataset that gave the most robust (in the sense that in most cases the results are better than results from using genotyped sample) and improved (in the sense that in most cases the results are better than results from using other filtering thresholds) results at the 8 SNPs to conduct GWAS of Alzheimer disease, fibrinogen, HDL and uric acid adjusting for the same covariates with imputation status as an additional covariate to account for the potential phenotypic difference between genotyped and imputed samples.

## Supporting Information

Figure S1 **–log_10_(p-value) plot of rs4420638 at various certainty thresholds for Alzheimer disease.**
(TIFF)

Figure S2 **–log_10_(p-value) plot of rs4681 at various certainty thresholds for Fibrinogen.**
(TIFF)

Figure S3 **–log_10_(p-value) plot of rs3764261, rs1919484, rs10186236, rs1800588 at various certainty thresholds for HDL.**
(TIFF)

Figure S4 **–log_10_(p-value) plot of rs16890979, rs2231142, rs1165205 at various certainty thresholds for uric acid.**
(TIFF)

Figure S5 **Regional association plot of rs4420638 for Alzheimer disease using incorporated genotype data.**
(TIFF)

Figure S6 **Regional association plot of rs4681 for Fibrinogen using incorporated genotype data.**
(TIFF)

Figure S7 **Regional association plot of rs3764261 for HDL using incorporated genotype data.**
(TIF)

Figure S8 **Regional association plot of rs1919484 for HDL using incorporated genotype data.**
(TIF)

Figure S9 **Regional association plot of rs10186236 for HDL using incorporated genotype data.**
(TIF)

Figure S10 **Regional association plot of rs1800588 for HDL using incorporated genotype data.**
(TIF)

Figure S11 **Regional association plot of rs16890979 for uric acid using incorporated genotype data.**
(TIF)

Figure S12 **Regional association plot of rs2231142 for uric acid using incorporated genotype data.**
(TIF)

Figure S13 **Regional association plot of rs1165205 for uric acid using incorporated genotype data.**
(TIF)
