# Supplementary figures and images for: Using Family-Based Imputation in Genome-Wide Association Studies with Large Complex Pedigrees: The Framingham Heart Study
Source: PLoS One. 2012 Dec 17;7(12):e51589. doi: 10.1371/journal.pone.0051589 (PMC3524237; doi:10.1371/journal.pone.0051589)

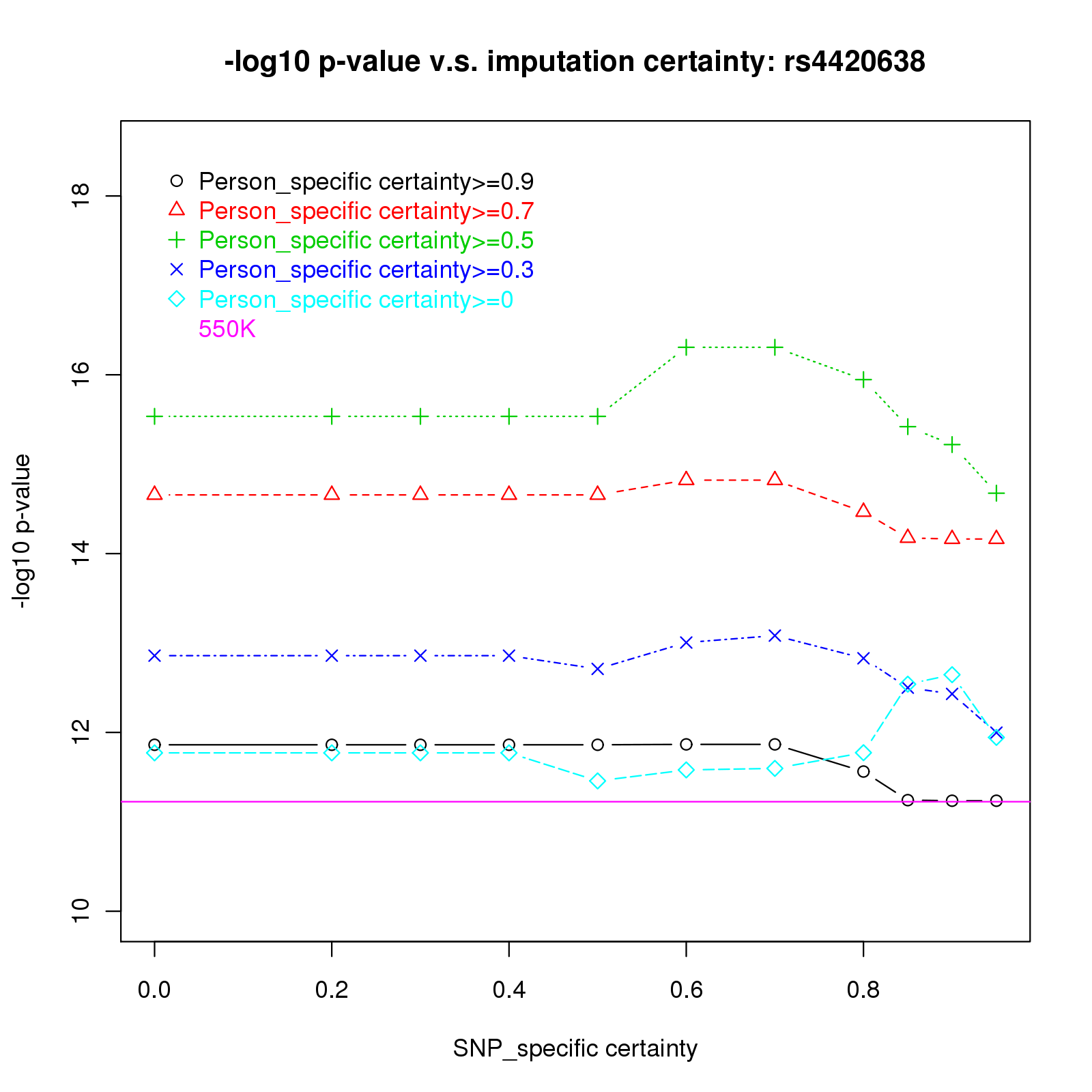

Supplement: Figure S1 — –log10(p-value) plot of rs4420638 at various certainty thresholds for Alzheimer disease. (TIFF) [file pone.0051589.s001.tiff]

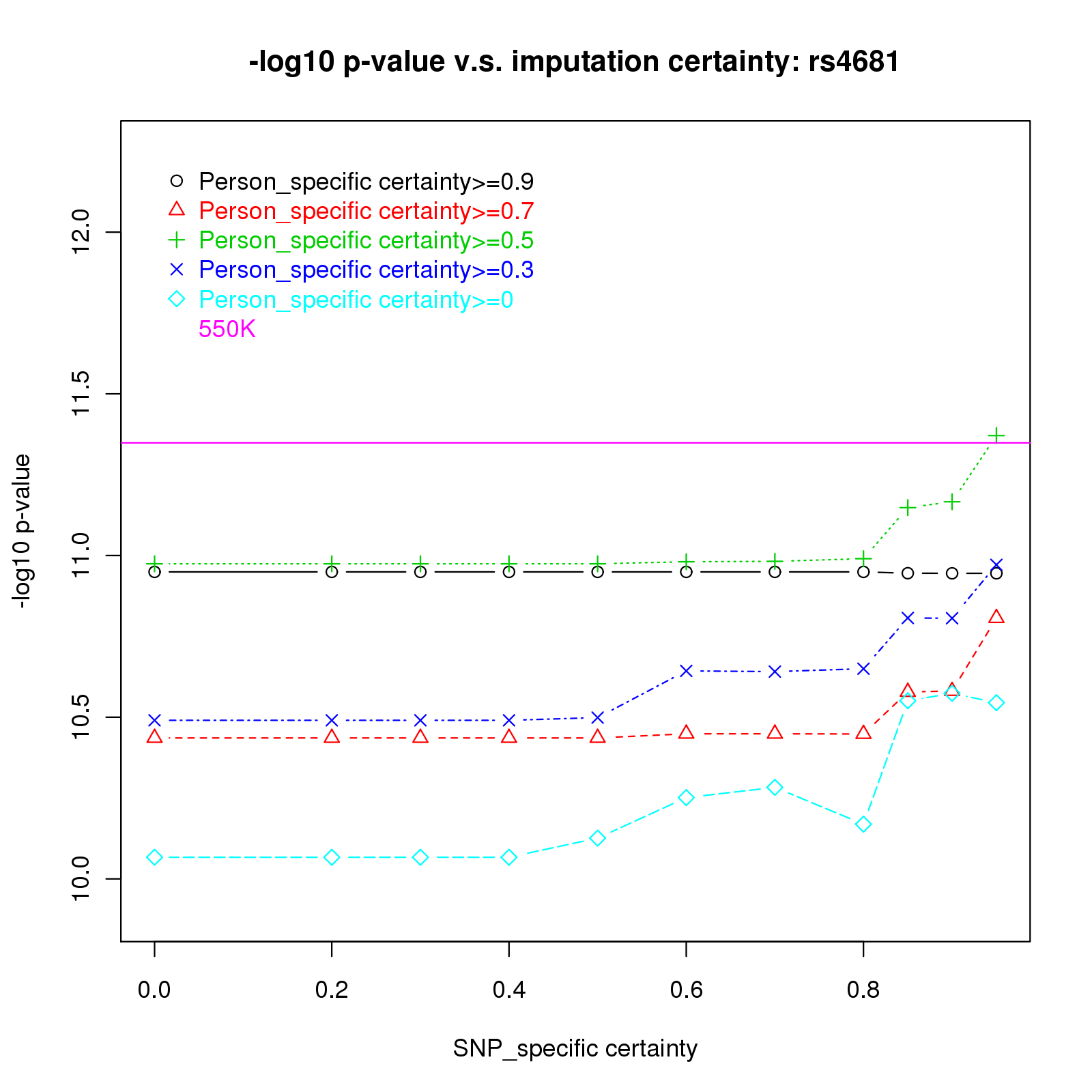

Supplement: Figure S2 — –log10(p-value) plot of rs4681 at various certainty thresholds for Fibrinogen. (TIFF) [file pone.0051589.s002.tiff]

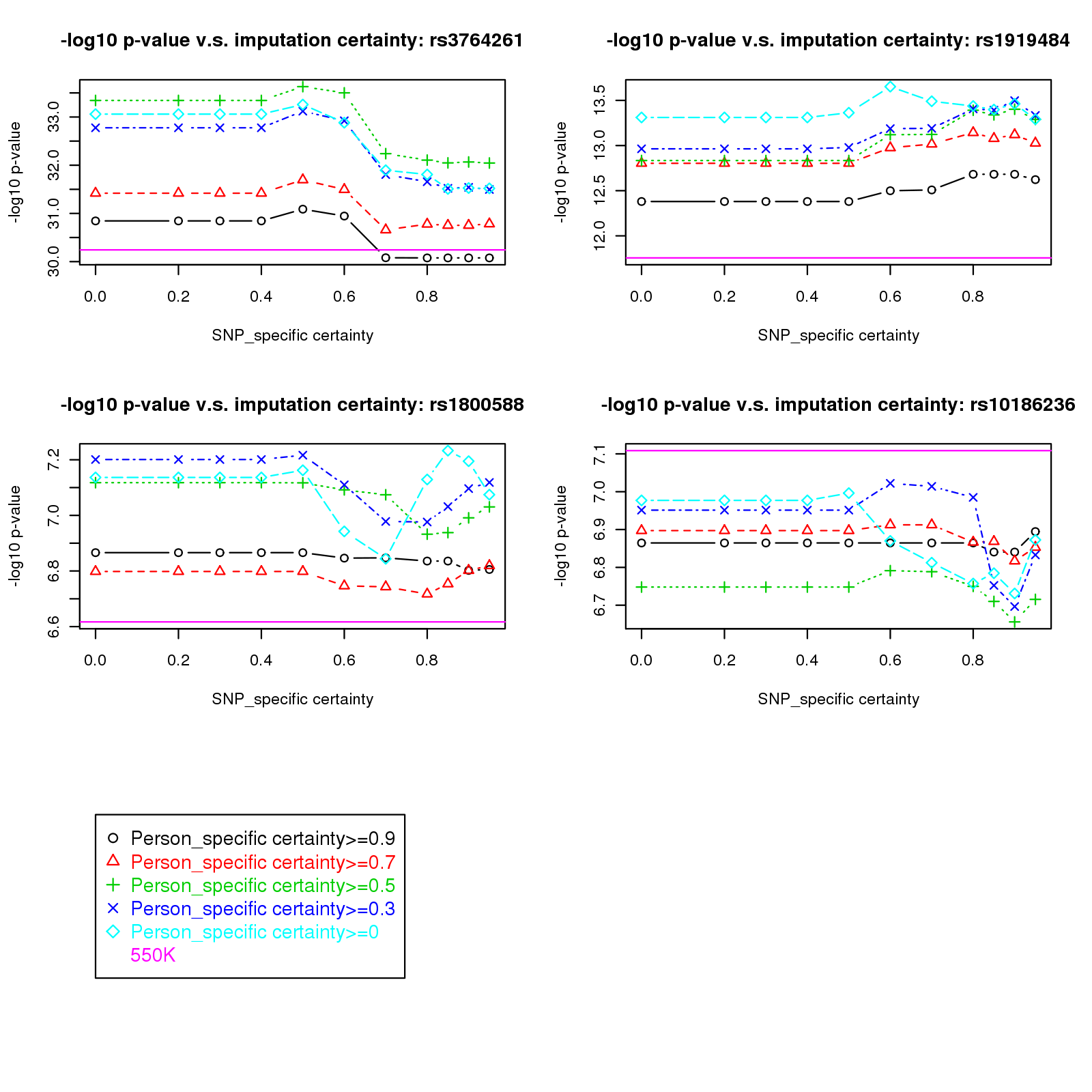

Supplement: Figure S3 — –log10(p-value) plot of rs3764261, rs1919484, rs10186236, rs1800588 at various certainty thresholds for HDL. (TIFF) [file pone.0051589.s003.tiff]

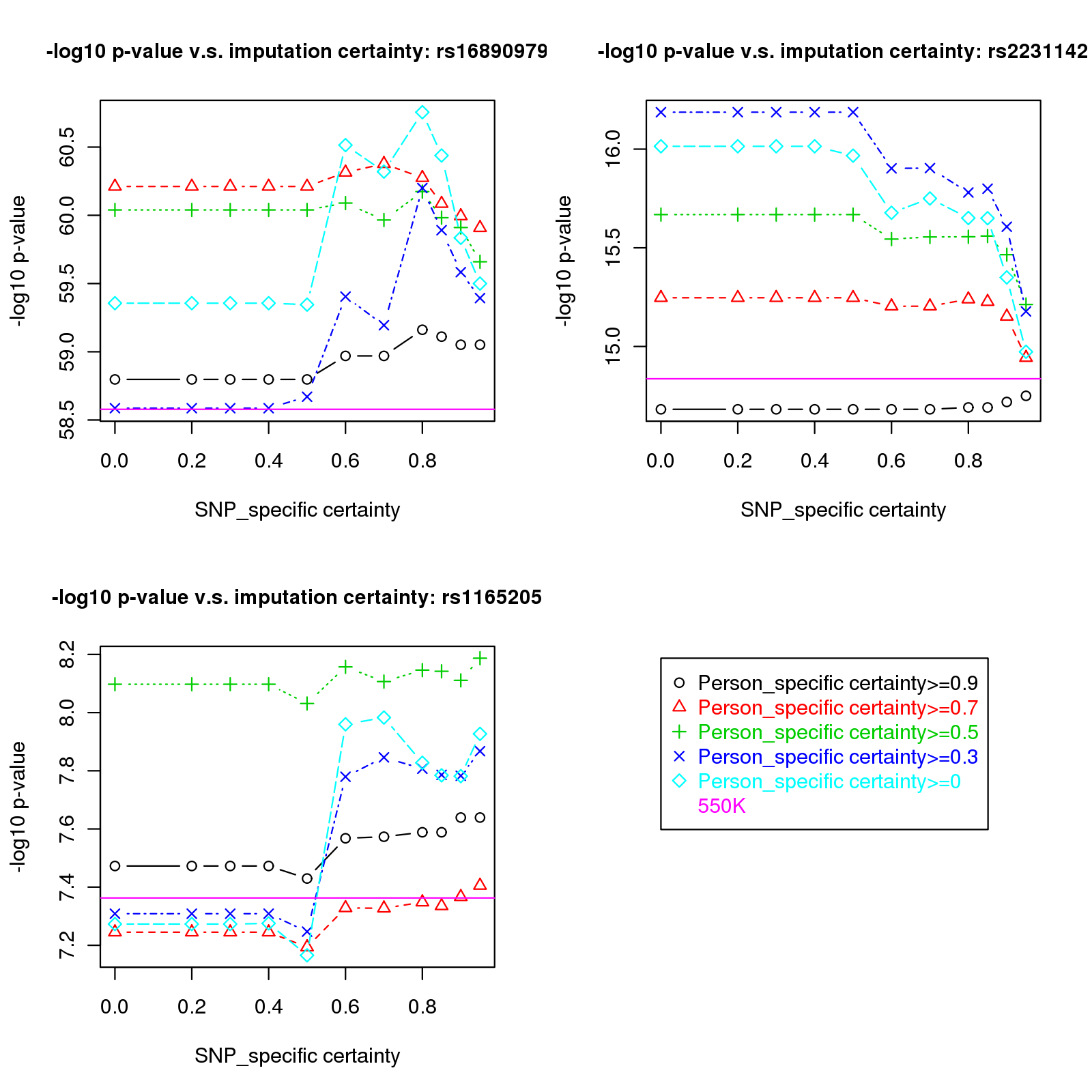

Supplement: Figure S4 — –log10(p-value) plot of rs16890979, rs2231142, rs1165205 at various certainty thresholds for uric acid. (TIFF) [file pone.0051589.s004.tiff]

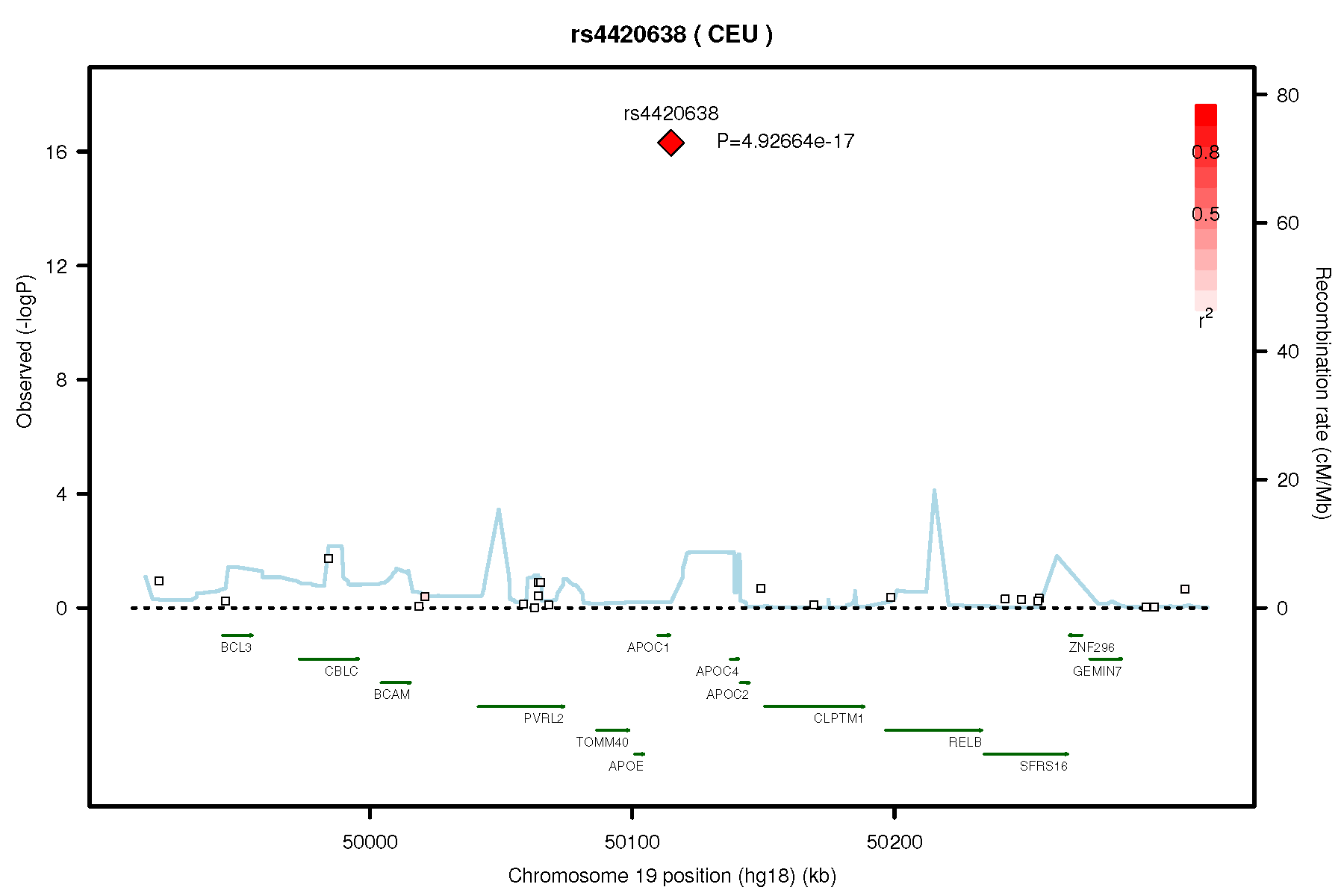

Supplement: Figure S5 — Regional association plot of rs4420638 for Alzheimer disease using incorporated genotype data. (TIFF) [file pone.0051589.s005.tiff]

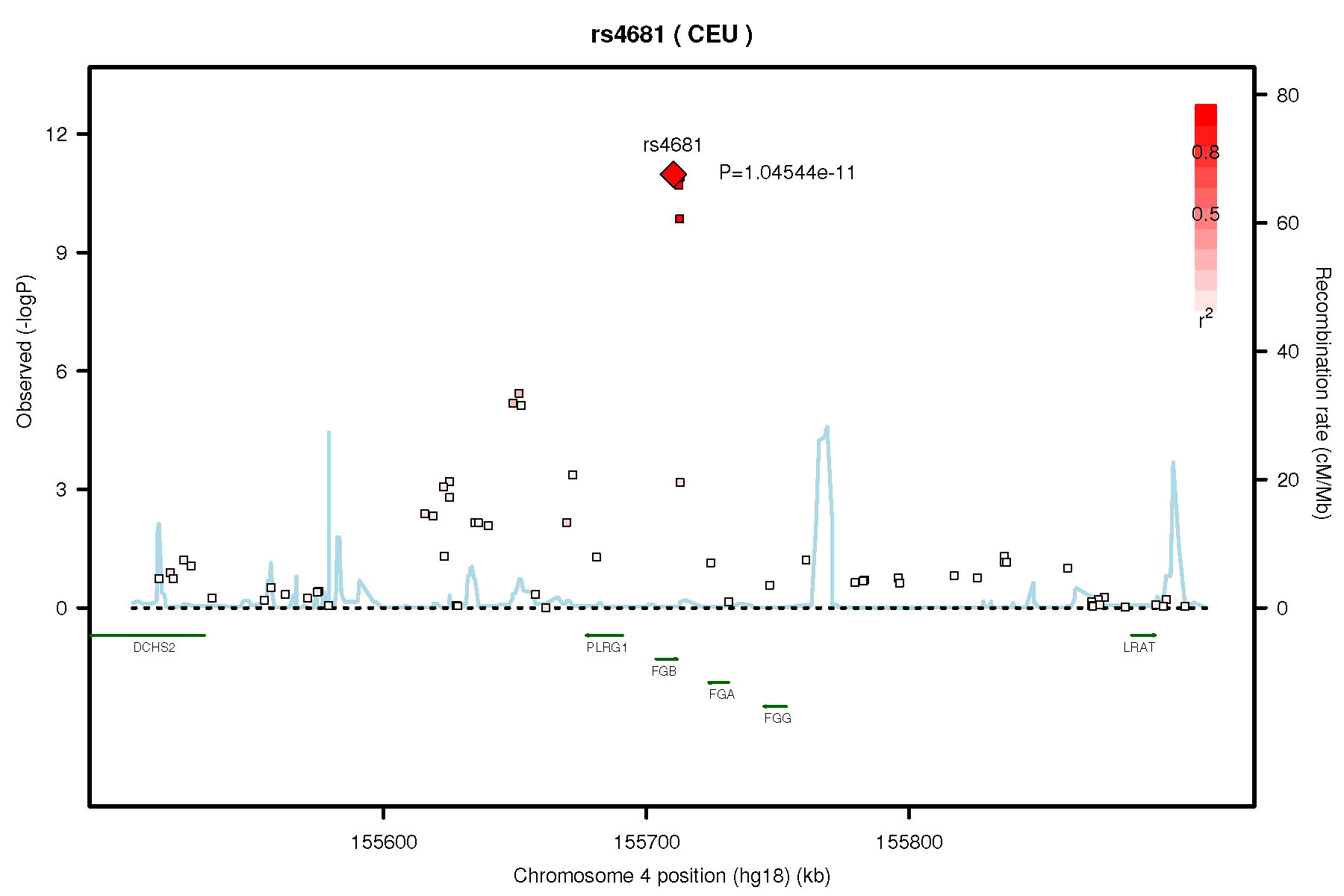

Supplement: Figure S6 — Regional association plot of rs4681 for Fibrinogen using incorporated genotype data. (TIFF) [file pone.0051589.s006.tiff]

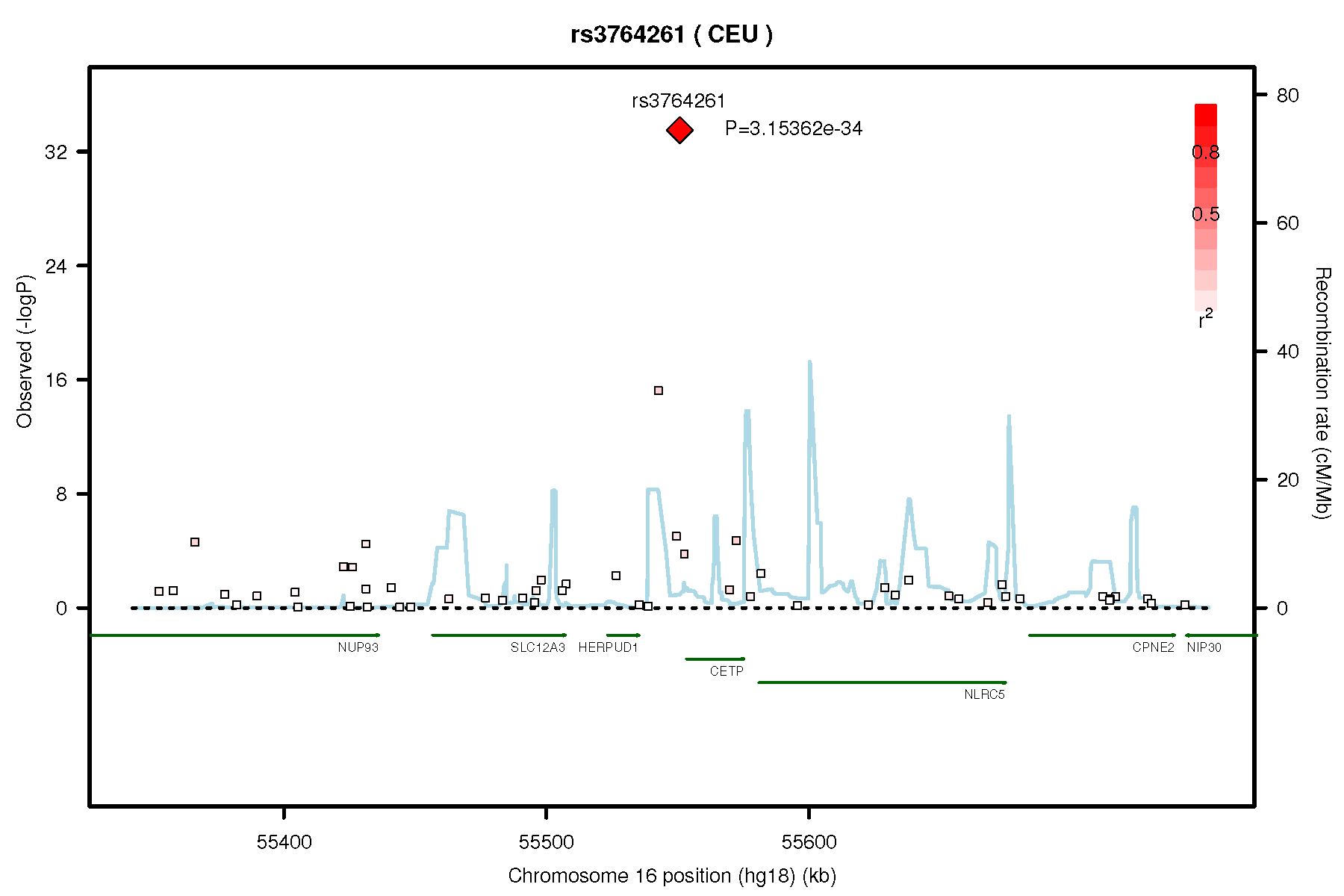

Supplement: Figure S7 — Regional association plot of rs3764261 for HDL using incorporated genotype data. (TIF) [file pone.0051589.s007.tif]

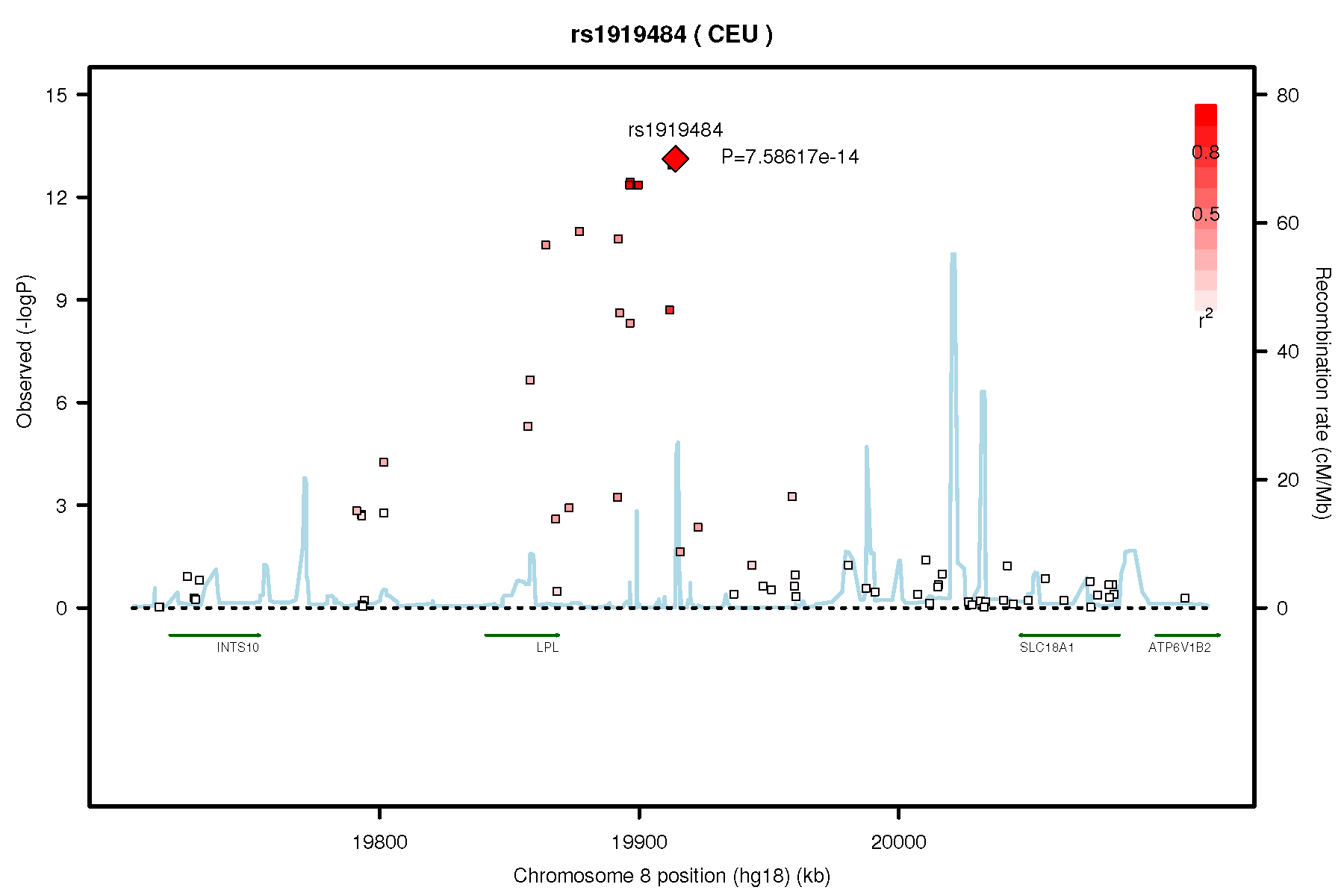

Supplement: Figure S8 — Regional association plot of rs1919484 for HDL using incorporated genotype data. (TIF) [file pone.0051589.s008.tif]

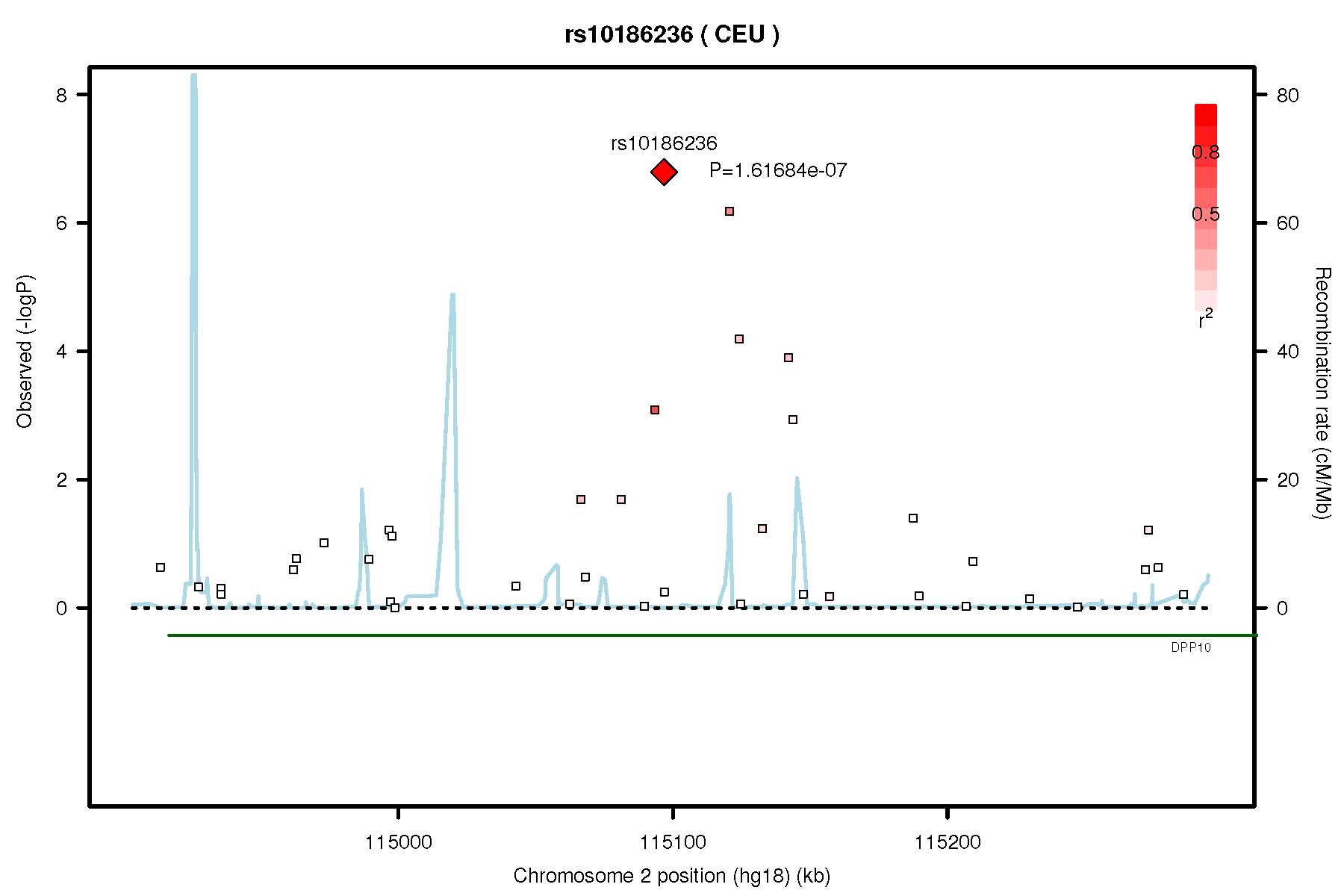

Supplement: Figure S9 — Regional association plot of rs10186236 for HDL using incorporated genotype data. (TIF) [file pone.0051589.s009.tif]

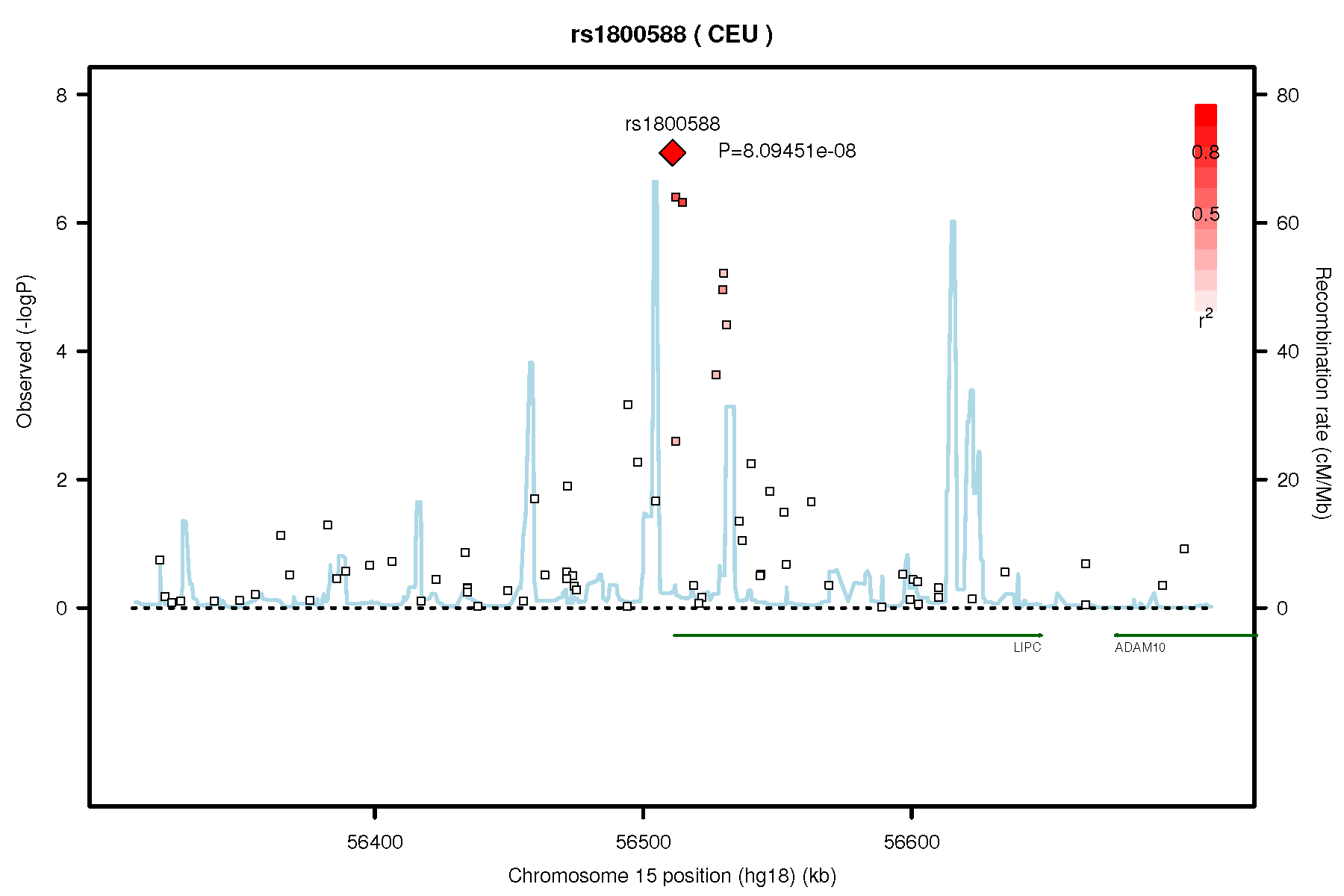

Supplement: Figure S10 — Regional association plot of rs1800588 for HDL using incorporated genotype data. (TIF) [file pone.0051589.s010.tif]

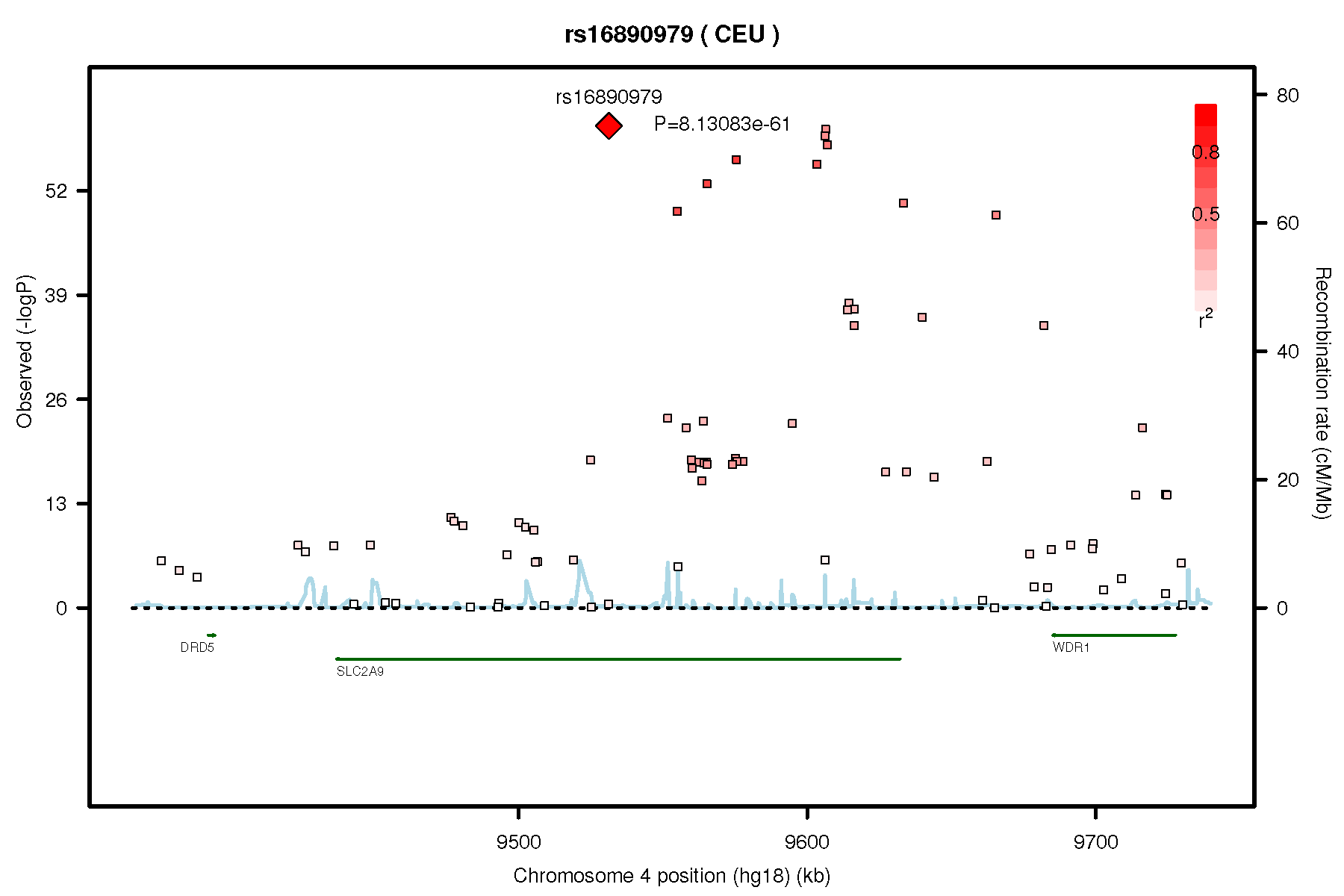

Supplement: Figure S11 — Regional association plot of rs16890979 for uric acid using incorporated genotype data. (TIF) [file pone.0051589.s011.tif]

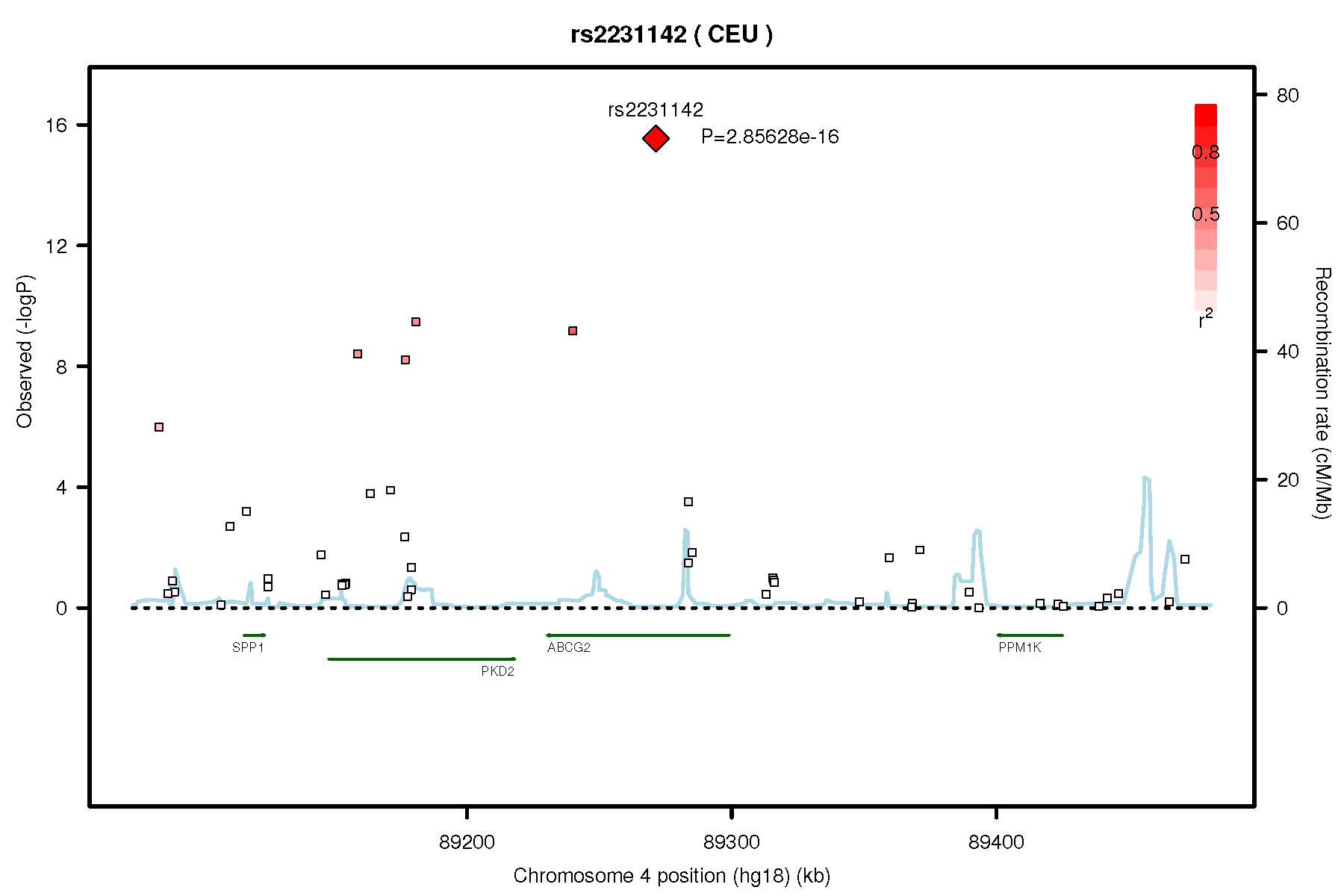

Supplement: Figure S12 — Regional association plot of rs2231142 for uric acid using incorporated genotype data. (TIF) [file pone.0051589.s012.tif]

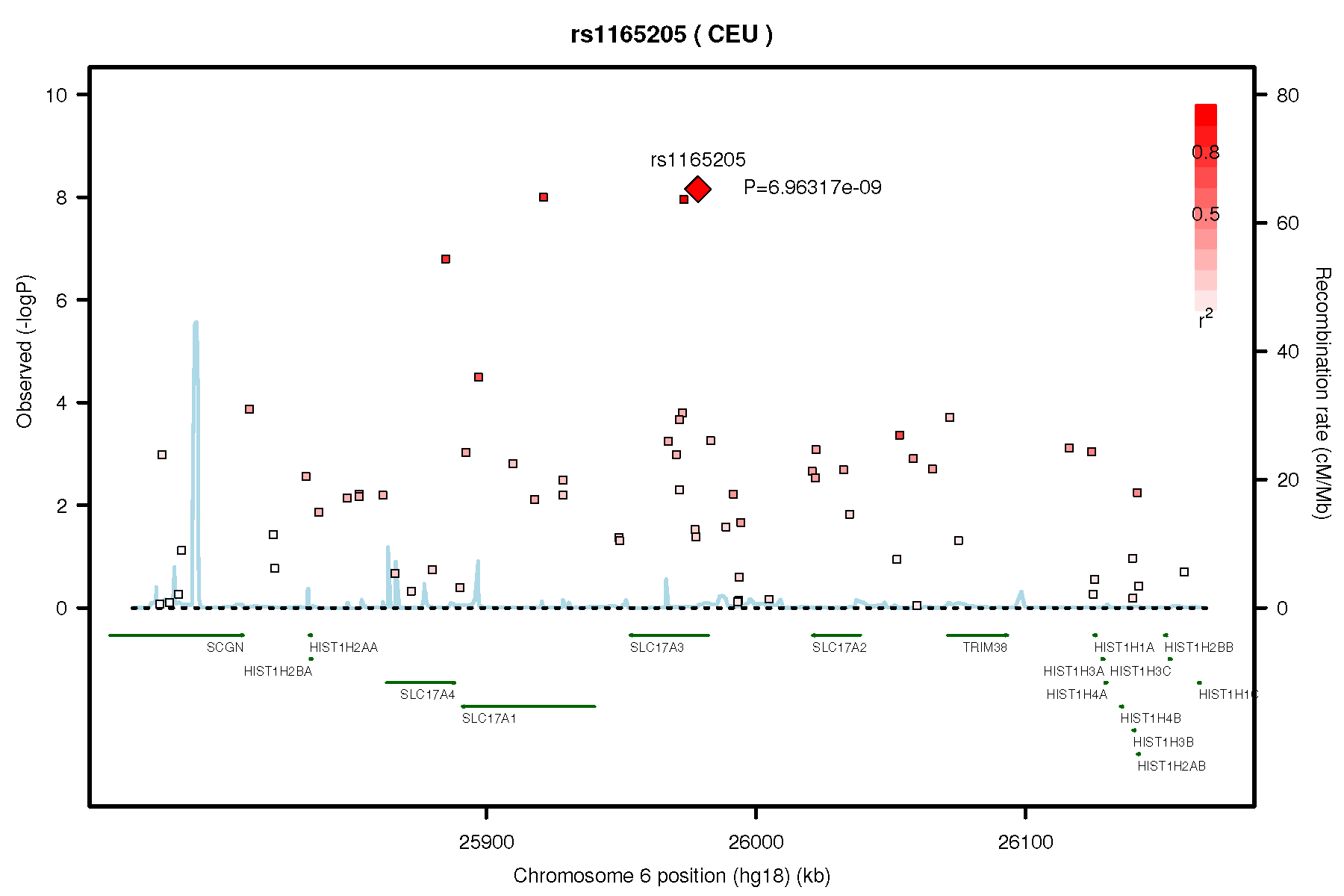

Supplement: Figure S13 — Regional association plot of rs1165205 for uric acid using incorporated genotype data. (TIF) [file pone.0051589.s013.tif]
